# Supplementary material for: Possibilities of an Electronic Nose on Piezoelectric Sensors with Polycomposite Coatings to Investigate the Microbiological Indicators of Milk
Source: Sensors (Basel). 2024 Jun 4;24(11):3634. doi: 10.3390/s24113634 (PMC11175303; doi:10.3390/s24113634)
Supplement: Supplementary file 1 [file sensors-24-03634-s001.zip › sensors-3013391-supplementary.pdf]

sensors

MDPI

Table S1. Specific mass sensitivity of sensor coatings to vapors of volatile compounds

Coating	Formic acid	Acetic acid	Butyric acid	Etha-nol	n-Butanol	Isobuta-nol	Iospenta-nol	Hexa-nol	Ace-tone	Buta-none-2	Acetalde-hyde	Ethylacet ate
18C6/Chitosan	0.09	1.08	26.42	0.14	0.54	2.43	1.70	0.99	0.07	1.55	0.42	0.29
DHC/Chitosan	0.85	8.70	1.10	0.18	0.23	2.55	4.04	2.85	0.03	1.77	0.85	1.05
Chitosan/CMC	0.29	5.32	5.03	0.11	0.39	0.05	1.26	1.25	0.05	0.24	0.61	0.52
Choline+Sorbitol	0.82	11.25	10.13	0.46	0.34	2.48	3.57	4.16	0.08	1.65	1.93	1.55
Choline+erythritol+ ASO	0.52	6.54	23.84	0.50	1.17	1.94	7.13	9.27	0.14	3.13	2.06	1.15
PVP/Chitosan	0.25	2.60	1.13	0.09	0.09	0.51	0.80	0.98	0.01	0.28	0.21	0.31
PEG2000/Chitosan	0.73	13.85	26.95	0.43	1.06	2.61	15.18	7.76	0.20	1.44	2.65	0.20
Erythritol+ASO	0.17	1.95	27.81	0.25	0.65	3.61	3.41	2.34	0.08	3.16	0.85	0.62

Table S2. Physical and chemical indicators of raw milk samples.

No	Mass fraction of dry solids. %	Mass fraction of fat. %.	Mass fraction of total protein. %	Density. kg/m³	Titratable acidity. ⁰T	% content of fat globules in the size of µm			Purity group
						0.01-10.00 µm	10.01-20.00 µm	>20.01 µm	
1	16.02±0.12	7.5±0.3	3.46±0.15	1025±0.5	19±0.5	99.89±0.030	0.07±0.005	0.04±0.003	I
2	12.22±0.13	3.8±0.1	3.74±0.10	1031±0.5	20±0.5	62.88±0.090	36.20±0.020	0.92±0.007	I
3	13.36±0.08	4.8±0.1	3.45±0.10	1032±0.5	19±0.5	82.83±0.040	17.16±0.009	-	I
4	15.15±0.14	7.5±0.5	3.26±0.10	1024±0.5	15±0.5	82.55±0.025	9.76±0.008	7.69±0.004	I
5	11.63±0.13	3.5±0.1	3.01±0.10	1028±0.5	19±0.5	89.17±0.031	10.83±0.009	-	I
6	11.77±0.11	3.1±0.1	3.30±0.15	1030±0.5	19±0.5	87.26±0.024	12.74±0.006	-	I
7	10.83±0.09	3.9±0.1	2.40±0.10	1030±0.5	15±0.5	99.94±0.021	0.06±0.0017	-	I
8	12.31±0.12	3.7±0.1	3.10±0.15	1027±0.5	18±0.5	75.33±0.037	23.79±0.009	0.88±0.005	I
9	11.41±0.06	3.2±0.1	2.00±0.05	1028±0.5	15±0.5	93.74±0.042	6.26±0.008	-	I
10	12.14±0.10	4.1±0.1	2.88±0.10	1028±0.5	16±0.5	100.00±0.010	-	-	I
11	11.72±0.07	3.4±0.1	1.16±0.10	1028±0.5	15±0.5	100.00±0.008	-	-	I
12	10.92±0.09	3.3±0.1	1.35±0.10	1027±0.5	11±0.5	100.00±0.009	-	-	I
13	11.44±0.11	3.6±0.1	2.59±0.15	1028±0.5	17±0.5	100.00±0.011	-	-	I
14	15.07±0.15	6.5±0.3	3.07±0.10	1026±0.5	16±0.5	99.77±0.022	0.18±0.003	0.05±0.002	I

sensors

MDPI

Table S3. Regression coefficients for variables of the models to predict the microbiological indicators of milk.

Prediction of QMAFAnM, lg(CFU/cm³)

No Factor	7#1	21#1	39#1	40#2	59#2	41#3	22#4	18#5	20#5	4#6	43#6	48#6	61#6	75#6	30#7	34#7	39#7	42#7	47#7
1	-0.81	9.13	-5.96	24.44	54.32	6.84	56.59	-5.82	-21.55	-0.25	6.72	-5.01	-13.81	-4.81	14.64	6.04	26.19	-20.45	8.12
2	-1.56	9.45	-11.19	36.15	59.91	11.64	42.30	-6.22	-21.55	0.45	5.59	-4.81	-14.43	-7.58	16.63	8.24	29.75	-22.38	11.18
3	-1.59	9.29	-16.11	39.54	64.22	12.64	46.70	-13.85	-19.29	0.91	4.62	-5.63	-14.26	-8.97	17.02	12.76	28.70	-21.77	16.92

	50#7	30#8	68#8	Am1.1	Am1/1.2	Am80.3	Am.3/1.4	Am1.8	Fmax.1	A7.1/11.2	Fmax.3	A4/22(4)	A4.4/5.20	Fmax.5	Fmax.6	Fmax.7	Fmax.8	A43∑	A68∑
1	-17.85	-6.00	12.89	0.16	0.01	0.01	0.04	0.07	0.12	0.09	0.16	0.20	0.19	-0.24	-0.28	-0.22	-0.54	0.0094	0.0022
2	-16.10	-8.18	14.13	0.23	0.01	0.01	0.06	0.13	0.23	0.11	0.21	0.13	0.12	-0.21	-0.30	-0.27	-0.49	0.016	0.0016
3	-16.27	-10.40	12.16	0.20	0.01	0.01	0.07	0.12	0.18	0.08	0.24	0.15	0.17	-0.13	-0.38	-0.37	-0.51	0.015	0.0017

Prediction of Yeast, lg(CFU/cm³)

	20#1	55#1	12#3	73#3	20#5	60#6	34#7	30#8	58#8	Am1.12	Am80.2	Am12.2	Am1/1.2	Am12.8	Fmax.1	A7.1/11.2	A11/40(2)	A11/59(2)	Fmax.6
1	-7.98	-8.81	4.30	5.23	-13.01	-8.93	-5.71	4.33	-7.35	-0.02	0.06	0.06	-0.03	0.09	-0.12	0.06	0.29	0.23	-0.32

	A37.6/15.7	Fmax.7	A6/30(8)	A43∑	A45∑	A68∑
1	-0.43	-0.33	0.04	-0.02	-0.19	0.0033

Prediction of Mold, lg(CFU/cm³)

	20#1	21#1	39#1	42#1	11#2	4#4	22#4	4#6	43#6	61#6	20#7	30#7	34#7	30#8	Am12.1	Am12.2	Am1/1.2	Am1.3	Am80.3
1	10.00	5.91	-6.16	10.40	3.08	0.24	-70.01	0.72	3.14	6.74	9.33	10.43	11.45	-5.94	0.07	0.10	0.04	0.05	0.01
2	12.26	10.23	-7.99	13.10	2.84	0.23	-75.44	0.61	3.88	8.01	9.26	13.01	11.00	-7.84	0.09	0.12	0.03	0.04	0.01

	Am12.3	Am80.6	Am12.6	Am80.7	Am7/1.8	A11/40(2)	A11/59(2)	A22.4/37.6	Fmax.5	Fmax.6	Fmax.7	A43∑	A45∑
1	0.11	0.05	0.16	0.02	0.04	-0.22	-0.05	-0.20	0.16	0.25	0.39	0.02	0.14
2	0.05	-0.22	-0.04	-0.23	0.16	0.24	0.36	0.02	0.15	0.05	-0.22	-0.04	-0.23
